# Roadmap for HCC Surveillance and Management in the Asia Pacific

**DOI:** 10.3390/cancers17121928

**Published:** 2025-06-10

**Authors:** Masatoshi Kudo, Bui Thi Oanh, Chien-Jen Chen, Do Thi Ngat, Jacob George, Do Young Kim, Luckxawan Pimsawadi, Pisit Tangkijvanich, Raoh-Fang Pwu, Rosmawati Mohamed, Sakarn Bunnag, Sheng-Nan Lu, Sirintip Kudtiyakarn, Tatsuya Kanto, Teerha Piratvisuth, Chao-Chun Wu, Roberta Sarno

**Affiliations:** 1 Department of Gastroenterology and Hepatology, Faculty of Medicine, Kindai University, Osaka 589-8511, Japan; m-kudo@med.kindai.ac.jp; 2 Department of Epidemiology, National Cancer Hospital, Hanoi 100000, Vietnam; buioanh1310@gmail.com; 3 Genomics Research Center, Academia Sinica, Nangang District, Taipei City 115, Taiwan; chencj@gate.sinica.edu.tw; 4 Medical Profession & Officer Health Protection Division, Department of Medical Service Administration, Vietnam Ministry of Health, Hanoi 11110, Vietnam; 5 Storr Liver Centre, Westmead Institute for Medical Research, University of Sydney, Sydney, NSW 2145, Australia; jacob.george@sydney.edu.au; 6 College of Medicine, Yonsei University, 50-1 Yonsei-ro, Seodaemun-gu, Seoul 03722, Republic of Korea; dyk1025@yuhs.ac; 7 Liver Cancer Patient Group, Bangkok 10110, Thailand; luckxawan.p@bu.ac.th; 8 Faculty of Medicine, Chulalongkorn University, Bangkok 10330, Thailand; 9 Data Science Center, Fu Jen Catholic University, New Taipei City 24205, Taiwan; 10 Faculty of Medicine, Universiti Kebangsaan Malaysia, Kuala Lumpur 43600, Selangor, Malaysia; ros@ummc.edu.my; 11 Department of Medical Services, Ministry of Public Health, Nonthaburi 11000, Thailand; sakarn.b@dms.mail.go.th; 12 Department of Internal Medicine, Kaohsiung Medical University Hospital, Kaohsiung 80708, Taiwan; juten@ms17.hinet.net; 13 Thai Cancer Society, Bangkok 10220, Thailand; sirintip@thaicancersociety.com; 14 Department of Hepatology, National Center for Global Health and Medicine, Tokyo 162-8655, Japan; kanto.t@jihs.go.jp; 15 NKC Institute of Gastroenterology and Hepatology, Songklanagarind Hospital, Prince of Songkla University, Hat Yai 90110, Thailand; teerha.p@psu.ac.th; 16 Health Promotion Administration, Ministry of Health and Welfare, Taipei City 103, Taiwan; 17 APAC Liver Disease Alliance, D-Health Consulting Pte. Ltd., Singapore 068914, Singapore

**Keywords:** hepatocellular carcinoma, Asia-Pacific, HCC awareness, HCC prevention, HCC early detection, healthcare systems, HCC surveillance, HCC treatment access, HCC surveillance and management, regional healthcare challenges, policy

## Abstract

**Background/Objectives:** Hepatocellular carcinoma (HCC) is a leading cause of cancer-related mortality, with the Asia-Pacific (APAC) region bearing a disproportionate burden. This paper examines HCC challenges within seven APAC health systems, identifies key barriers at each stage of the patient journey, and proposes tailored, actionable solutions. To effectively address HCC challenges, a stepwise approach should prioritise high-impact solutions, focusing on prevention, early diagnosis, and expanding surveillance to maximise health outcomes and economic benefits, while tailoring strategies to each health system’s unique resources and constraints. **Methods:** A mixed-methods approach was used, including expert consultations from the 2024 HCC APAC Policy Forum, a literature review, and a review of Japan’s HCC management model. Data were collected through workshops and stakeholder feedback from healthcare professionals, policymakers, researchers and patient advocates across Australia, India, Malaysia, South Korea, Taiwan, Thailand, and Vietnam. **Results:** Key findings include significant disparities in HCC awareness, prevention, early detection, diagnosis, and access to treatment. Common challenges across APAC include limited public awareness, suboptimal surveillance infrastructure, and financial barriers to care. The integration of novel biomarkers and advanced surveillance modalities were identified as crucial priorities for improving early detection. Japan’s multi-faceted approach to HCC management serves as a successful model for the region. **Conclusions:** A customised and targeted approach is essential for reducing the HCC burden across APAC. The proposed recommendations, tailored to each health system’s needs, can significantly improve patient outcomes and reduce healthcare costs. Effective collaboration among stakeholders is necessary to drive these changes.

## Simple Summary

Hepatocellular carcinoma (HCC) is a major health issue in the Asia-Pacific region, where the number of cases is rising rapidly. Many people are diagnosed at later stages, making it harder to treat and manage. This roadmap examines the challenges people face across seven health systems in detecting and treating liver cancer, such as limited awareness, lack of robust surveillance, and financial barriers. The authors aim to identify solutions tailored to the needs of different countries, drawing from successful approaches like those used in Japan. By improving awareness, expanding access to early detection, and ensuring more affordable treatments, the research hopes to reduce the burden of liver cancer and improve the quality of life for those affected. The findings could provide valuable guidance for healthcare systems working to combat liver cancer.

## 1. The Need to Act on Hepatocellular Carcinoma

Primary liver cancer is a major global health challenge, with hepatocellular carcinoma (HCC) accounting for 85–90% of cases [1,2]. HCC primarily affects individuals with chronic liver conditions such as cirrhosis caused by hepatitis B (HBV) or hepatitis C (HCV) infections [2]. However, non-viral risk factors–including obesity, diabetes, Metabolic Dysfunction-Associated Steatotic Liver Disease (MASLD; formerly NAFLD), alcohol-related liver disease, and aflatoxin exposure–are increasingly contributing to the disease burden [3,4].

### 1.1. Urgent Action Is Needed in Asia Pacific, Which Shoulders a Disproportionate Share of the Global Liver Cancer Burden

In 2022, an estimated 866,136 new cases of liver cancer were diagnosed globally, causing 758,725 deaths [5]. The APAC region carries 73% of the global HCC burden [6]. Although HBV and HCV account for the majority of HCC cases, significant progress in addressing these infections has led to a relative decline in viral hepatitis-related HCC [7]. In 1990, HBV accounted for over half of all HCC cases, declining to 42% by 2019 [2]. At the same time, the incidence of MASLD has risen by an average of 0.21% per year from 2010 to 2019 [8]. This trend signals a growing shift towards non-viral aetiologies. This transition underscores the urgent need to address the increasing burden of MASLD-induced HCC, which is being driven by rising obesity and type 2 diabetes rates, particularly among the younger demographic [2]. Unhealthy lifestyles, such as poor diets and sedentary behaviours, are also on the rise, leading to a growing prevalence of metabolic risk factors across APAC [9].

This evolving risk profile demands a multi-faceted response that not only targets traditional viral causes (i.e., HBV, HCV) but also accounts for metabolic (i.e., Obesity, diabetes, MASLD), immune-related (i.e., Primary biliary cholangitis, primary sclerosing cholangitis and autoimmune liver disease), and toxicological (i.e., Alcohol consumption, aflatoxin exposure, pesticide exposure) contributors to HCC [2,3].

### 1.2. Strengthening Surveillance and Management of Hepatocellular Carcinoma in Asia Pacific Can Significantly Reduce Hepatocellular Carcinoma Burden on Healthcare Systems, Societies and Economies

Despite progress, gaps persist in early detection and timely intervention for HCC, leading to suboptimal patient outcomes. Over 80% of HCC cases in APAC are diagnosed at advanced stages, limiting curative treatment options and making HCC the second leading cause of premature cancer mortality [10]. The economic implications of HCC in APAC are equally concerning. In China alone, liver cancer costs USD 11.1 billion annually (0.047% of national health spending), a figure expected to rise 206% to USD 34.0 billion by 2030 [11].

Implementing robust HCC surveillance and management systems is critical to alleviating the significant burden HCC places on healthcare systems, societies, and economies across APAC. HCC surveillance involves systematic efforts to identify individuals at risk of developing HCC or detecting the disease at its earliest stages through regular monitoring, while HCC management encompasses a continuum of care that includes early diagnosis, timely access to effective treatment, and ongoing patient support to improve outcomes and quality of life.

A structured roadmap for HCC surveillance and management, tailored to each APAC health system’s unique challenges and capacities, is essential to drive early detection and timely treatment for HCC. Achieving this requires collaboration among policymakers, healthcare providers, researchers, and patient advocates to ensure that solutions are practical, sustainable, and equitable.

### 1.3. While the Challenges Facing Hepatocellular Carcinoma Are Significant, the Opportunity to Significantly Reduce the Overall Burden Is Within Reach

HCC can be diagnosed early through surveillance methods such as imaging and biomarker testing, allowing for timely and effective treatment [12]. Early diagnosis enables curative treatment options like surgical resection, liver transplantation, or locoregional therapies, which significantly improve patient outcomes and reduce the strain on healthcare systems. The urgency to act cannot be overstated. Elevating HCC as a public health priority and implementing tailored, actionable surveillance and management strategies can reduce mortality and economic burden. A unified, region-wide approach that strengthens prevention, surveillance, diagnosis, and treatment systems will improve outcomes for millions across the region. Japan provides a successful model, demonstrating that comprehensive awareness campaigns, early detection programmes, and effective treatment strategies can significantly reduce HCC incidence and mortality. However, each health system must adapt best practices to local structures, leveraging context-specific solutions. This will be explored in detail later.

## 2. A Methodology Grounded in Local Insights and Evidence

This paper presents actionable recommendations for improving HCC surveillance and management across seven APAC health systems: Australia, India, Malaysia, South Korea, Taiwan, Thailand, and Vietnam. These recommendations are based on local expert insights and supplemented with learnings from Japan’s success in managing HCC and a targeted literature search.

### 2.1. Local Expert Insights and Regional Collaboration Are the Cornerstones of Mitigating HCC Burden

The basis of this paper lies in the expertise and feedback gathered during the 2024 HCC APAC Policy Forum, organised by the APAC Liver Disease Alliance in Thailand. This forum convened over 70 stakeholders from seven health systems, including health ministry officials, clinicians, researchers, and patient advocates. Participants of the Policy Forum represent a diverse range of key stakeholder groups across each health system. The selection prioritised multidisciplinary perspectives, including public and private sectors, urban and rural settings, and various clinical specialities, to capture the full range of challenges and opportunities in HCC.

### 2.2. Supplementing Expert Insights with Targeted Literature Search

Expert recommendations were gathered through a structured, multi-step process during the 2024 HCC APAC Policy Forum workshops, which included facilitated group discussions, plenary sessions, and breakout working groups focusing on specific patient journey stages.

Data was recorded through detailed notes and audio recordings. A thematic analysis identified recurring challenges, priorities, and potential solutions. This enabled consolidation of findings and refinement of recommendations based on participant feedback, enhancing validity and relevance.

These insights were then contextualised through a targeted literature review focusing on scientific publications, national HCC action plans, and reports from regional and international organisations relevant to HCC surveillance and management in the APAC region. Key databases, including PubMed and Scopus, were searched using specific terms such as “hepatocellular carcinoma”, “HCC surveillance”, “HCC management”, “APAC”, and health system-specific keywords. The review primarily included studies published between 2015 and 2025 to capture recent advances, with selective inclusion of seminal works and foundational guidelines predating this period to provide essential context.

A key resource was the APAC Liver Disease Alliance’s 2023 white paper, “Eliminating Asia’s Silent Emergency: Hepatitis and Hepatocellular Carcinoma” which provided insights into HCC epidemiology, aetiology, surveillance, and management across 13 APAC systems [13].

### 2.3. Rationale for the Patient Journey Stage Framework

To systematically identify and address barriers along the continuum of care, we utilised the Patient Journey Stage framework, dividing the HCC care pathway into Awareness, Prevention, Early Detection, Diagnosis, and Access to Treatment stages. This original and comprehensive framework enables a detailed analysis of health system challenges, ensuring recommendations are tailored to specific patient experiences and interventions at each stage. It also facilitates targeted identification of gaps and supports stepwise prioritisation of solutions to maximise both health and economic outcomes.

### 2.4. Integration of Japan’s Roadmap as a Model of the World’s Best Practice

A key highlight of the workshop, “Co-creating a Roadmap for Robust National HCC Surveillance and Management Programmes in APAC”, was the adoption of Japan’s roadmap, structured around the patient journey framework, as a model. Japan’s best practices in HCC surveillance, early diagnosis, and management served as a catalyst for discussion and a foundational benchmark for this paper. Workshop experts analysed and contextualised Japan’s strategies, exploring how they could be adapted to diverse APAC health systems while accounting for differences in infrastructure, resources, reimbursement policies, and epidemiological profiles. This comparative approach ensures that the recommendations in this paper are both goal-oriented and feasible within diverse APAC contexts. As a leader in HCC surveillance, early diagnosis, and management, Japan has demonstrated how robust, evidence-based roadmaps, supported by awareness, technology integration, and comprehensive reimbursement can significantly reduce the burden of disease.

### 2.5. Limitations and Potential Biases

While this methodology draws on local expert insights and a comprehensive literature review, several limitations must be acknowledged. The expert sample, though multidisciplinary and geographically diverse, was drawn from a select group of participants at the 2024 HCC APAC Policy Forum and may not fully represent the full spectrum of perspectives across the seven APAC health systems. Consequently, the generalisability of the recommendations is context-dependent and should be viewed as a foundational starting point, to be refined through broader stakeholder engagement. Additionally, the targeted literature review may have excluded relevant unpublished studies or non-English language publications, potentially limiting comprehensiveness. These factors underscore the importance of ongoing validation and iterative updates to the roadmap as new evidence and perspectives arise.

## 3. Learning from Japan: A World-Leading Case Study

Japan has established a comprehensive and highly structured approach to HCC surveillance and management (see Table 1), serving as a global benchmark for healthcare systems worldwide. Through targeted interventions that encompass proactive prevention, early detection, timely diagnosis, and effective, affordable and equitable access to care, Japan has successfully addressed critical challenges across the entire patient journey [14]. By combining public health initiatives, financial support, and advanced medical technologies, Japan’s model can inspire healthcare improvements across the APAC region.

Japan’s multi-faceted strategy focuses on raising awareness, strengthening surveillance and early detection and expanding healthcare access. In 2024, the government allocated ¥16.8 billion (USD 108 million) to support early detection, treatment subsidies, and public awareness campaigns [17]. This achievement has been realised through collaboration between academic societies, patient advocacy groups, and policymakers. While this investment represents less than 1% of Japan’s annual national healthcare expenditure, it highlights how a relatively modest and strategic investment can lead to far-reaching, positive outcomes [18].

To facilitate timely diagnosis and referral, an integrated electronic medical records system automatically triggers specialist referrals for patients with positive viral hepatitis test results [19]. Japan also reimburses bi-annual surveillance and diagnostic tests, including biomarkers such as AFP, PIVKA-II, and AFP-L3, alongside ultrasound, CT, and MRI scans [20]. PIVKA-II, in particular, is highly specific to HCC, offering enhanced diagnostic accuracy and early detection capabilities, which are crucial for improving patient outcomes [21]. Its overall sensitivity and specificity in detecting HCC have been reported to range from 48% to 62% and 81% to 98%, respectively [21]. Japan’s early adoption of advanced surveillance modalities, such as PIVKA-II in 1989 and AFP-L3 in 1994, has enhanced cost-effective and simultaneous biomarker testing, improving early disease identification [19]. Surveillance protocols recommend routine ultrasound and biomarker assessments every six months for high-risk individuals (individuals with HBV, HCV, and non-viral cirrhosis), with more frequent monitoring for those at extremely high risk (i.e., individuals with cirrhosis caused by HBV or HCV C) [20]. Notably, 68% of HCC cases are detected at an early stage, demonstrating the system’s effectiveness [19]. Comprehensive cancer care networks further support patients throughout their treatment journey, contributing to improved survival rates, with median survival reaching 79.6 months for those undergoing regular surveillance [20]. This contrasts with a median survival of 20.9 months for patients in APAC [20,22].

The impact of Japan’s efforts is also evident in the steady decline of HCC mortality and the reduction in the overall cost of illness. In 2002, Japan recorded 34,637 HCC-related deaths with a mortality rate of 27.4 per 100,000 population, which decreased to 24,082 deaths in 2021 [19]. Recent studies show a decline in HCC incidence in Japan, primarily due to effective HCV treatments, with projections indicating a continued reduction in social burden and mortality until 2029 at an annual rate of 2.2% [23,24]. The cost of illness also trended downwards, with a decrease of 33%, with the presence of a national surveillance programme [17]. This highlights the economic benefits of a well-established HCC surveillance and management programme.

These improvements are attributed to effective surveillance programmes, widespread surveillance for HBV and HCV, and the adoption of advanced antiviral treatments [25]. By reducing new HCC cases and improving early detection rates, Japan has demonstrated the effectiveness of its multi-pronged strategy.

Japan’s comprehensive approach to HCC surveillance and management offers valuable insights that can be adapted and tailored to the unique healthcare landscapes of other APAC health systems. Japan’s approach and its applicability to other health systems depend on factors such as healthcare coverage, screening adherence, and investment in early detection. Tailoring HCC surveillance and management strategies to local infrastructure will maximise economic and public health benefits while ensuring sustainable long-term improvements.

## 4. Hepatocellular Carcinoma Challenges Facing Health Systems in the Asia Pacific

This section explores the persistent challenges faced by seven APAC health systems in implementing strategies aligned with Japan’s model. It maps key obstacles at each stage of the patient journey awareness and prevention to early detection, diagnosis, and access to treatment based on insights from local experts. These obstacles contribute to delays in diagnosis and suboptimal care, which can lead to higher mortality rates and a greater burden on the healthcare system.

In Australia, Indigenous and culturally and linguistically diverse (CALD) populations remain disproportionately affected by HCC, with obesity, diabetes, and alcohol-related liver disease contributing to rising incidence rates [26]. Hepatitis diagnosis and treatment uptake among younger people with newly acquired infections (typically people who inject drugs) also remains low (see Table 2) [27].

Early detection is challenging, with suboptimal surveillance participation despite established clinical guidelines. This is due to a combination of patient, clinician and system-level barriers [28]. Firstly, Indigenous Australians have a higher incidence of HCC and late-stage disease at diagnosis, and poorer survival, which may stem from reduced access to surveillance in addition to socio-environmental inequality, cultural barriers, and a distrust of the health care system [28]. Secondly, the Australian 2023 HCC Surveillance Guidelines recommend ultrasound surveillance for HCC with or without α-fetoprotein (AFP) [29]. However, the sensitivity of AFP and ultrasound combination in detecting early-stage HCC is suggested to be 60%, meaning that 40 out of 100 patients may not receive an early diagnosis despite undergoing surveillance [30,31]. Lastly, despite high surveillance rates reported among patients in tertiary liver clinics in Melbourne, data on HCC surveillance in primary care remains limited, and only 27% of patients show good adherence to HCC surveillance [28].

Ensuring equitable access to treatment is a challenge, as Indigenous Australians face 2.4 times higher incidence and mortality rates than non-Indigenous Australians [32]. Limited healthcare access in remote areas exacerbates these disparities, leading to preventable deaths [33]. Delayed treatment not only worsens patient outcomes but also increases healthcare costs due to prolonged hospitalisations, emergency care, and palliative care expenses [34].

HCC is a leading cause of cancer-related mortality in India [35]. Despite recent advances in early diagnosis and treatment, barriers persist in the patient’s journey to effective care (see Table 3). The first barrier is the lack of effective interpersonal communication to scale up awareness of HCC and its associated risk factors. While India has made strides in promoting awareness of hepatitis–one of the top risk factors of HCC–through the National Viral Hepatitis Control Programme [36], there is a rising incidence of HCC related to alcohol and MASLD [37].

A major issue in the prevention of HCC is the lack of affordable Re-Use Prevention (RUP) syringes. These play a key role in preventing HCV transmission and hence HCC alongside antiviral treatment and active surveillance of hepatitis [42]. These preventive efforts have been rolled out across the state of Punjab but have yet to be scaled up at a national level as part of the National Viral Hepatitis Control Programme [42].

Another challenge is a lack of widespread early detection and diagnosis. While Indian National Association for the Study of the Liver (INASL) guidelines suggest six-monthly HCC surveillance using abdominal ultrasound with or without AP testing, HCC surveillance is not well-organised or universally practised across India [41,43]. Geographic diversity adds to this challenge, with disparities in healthcare access hindering the implementation of HCC prevention and surveillance. This means that HCC is often diagnosed at later stages, where curative treatment is not possible and the prognosis is poor [41].

Lastly, many treatment modalities, especially for late-stage HCC, are not accessible or affordable for a significant portion of the population [35]. As HCC is often diagnosed at a late stage, patients are often relegated to palliative care [35]. The geographic diversity and large population of India, and the high cost of treatments such as immunotherapy for late-stage HCC, underscore the importance of tackling challenges associated with prevention, awareness and early detection of HCC.

**Table 4:** HCC challenges in Malaysia.

PATIENT JOURNEY STAGE	CHALLENGE
**AWARENESS**	• Low awareness among HCPs outside of hepatologists and gastroenterologists • Low awareness among potential at-risk patients, with a need to better identify who falls into high-risk categories • Low awareness among laboratory personnel about the availability of relevant tests
**PREVENTION**	• Lack of screening for HCC risk factors (e.g., HBV, HCV, MASLD, and alcohol-related liver conditions) to prevent progression to HCC
**EARLY DETECTION**	• Lack of HCC surveillance for early detection of high-risk groups
**DIAGNOSIS**	• Diagnoses and management are not conducted within a multidisciplinary team setting, hindering access to care and treatment • Access to a multidisciplinary team approach needs to be strengthened (e.g., virtual multidisciplinary team discussion, to physically refer if there is a definitive management plan)
**ACCESS TO TREATMENT**	• Lack of access to evidence-based treatment options for patients

One of the primary challenges in Malaysia is the limited awareness among healthcare professionals and the general public (see Table 4) [44]. While hepatologists and gastroenterologists are well-versed in HCC, there is a need to increase awareness among other specialists and general practitioners [45]. This lack of awareness extends to the public, particularly those at high risk of HCC, who may not recognise their vulnerability and, as a result, may not seek medical advice until the disease has progressed significantly. Additionally, laboratory personnel, who are essential in the diagnostic process, may not always be up to date with the latest testing protocols, delaying diagnosis.

Although there are efforts to treat identified cases of HBV, HCV, MASLD, and alcohol-related liver diseases, preventive measures are not always consistently promoted or implemented. For example, despite the availability of vaccinations and antiviral treatments, many at-risk individuals remain undiagnosed or untreated [45]. The National Strategic Plan for HBV and HCV 2019–2023 aims to curb viral hepatitis through prevention, surveillance, and treatment, but its impact is limited by insufficient public engagement [46]. This situation highlights the need for more comprehensive and targeted prevention efforts to reduce the future burden of HCC.

Malaysia also faces challenges in ensuring regular HCC surveillance of high-risk groups. The national cancer surveillance programme is not always widely implemented, particularly in rural areas or among vulnerable populations. For example, a significant proportion of HCC cases in Malaysia are diagnosed at Stage IV when curative treatment options become more limited [47]. In Malaysia, the absence of a national liver cancer screening programme leads to variations in surveillance protocols and eligibility criteria for high-risk individuals [48]. This inconsistency may lead to delayed diagnoses. Current surveillance programmes cover only sonography and/or 1 biomarker for HCC surveillance, which limits the sensitivity and effectiveness of early detection [49].

The integration of a multidisciplinary team can be strengthened in some healthcare settings [50]. This is essential for accurate diagnosis, effective treatment planning, improved patient outcomes and cost reduction, as more complex and expensive interventions are required in the advanced stages of the disease.

While Malaysia offers curative options such as liver resection and transplantation at relatively lower costs than some other healthcare systems, these treatments are concentrated in specialised centres with limited capacity, thereby hindering access [51]. High demand and resource constraints result in long waiting lists, delaying access to potentially life-saving procedures.

Although South Korea implemented a national HCC surveillance programme for high-risk populations and introduced a national HCV surveillance programme in 2017, low public awareness still results in some patients being diagnosed at later stages, when treatment options are fewer and less effective (see Table 5) [52]. This delay also increases healthcare costs, placing a financial strain on both individuals and the national healthcare system.

Early detection is critical to reduce the HCC burden. South Korea’s surveillance primarily relies on two biomarkers (AFP and PIVKA II) [53,54], but more accurate methods are needed to improve early-stage detection and patient outcomes [55].

Another challenge is the limited reimbursement options for essential HCC treatments, which create additional financial barriers for patients [56]. These treatment access issues exacerbate the progression of the disease [57]. The financial strain on patients, in turn, leads to delays in treatment, further worsening health outcomes.

In Taiwan, three-fourths of HCC cases were positive for HBsAg or anti-HCV [58], highlighting the strong association between viral hepatitis and HCC. Despite the availability of a national HBV and HCV screening programme [59] and a reimbursed HCC screening programme for hepatitis patients under national health insurance, one-third of HCC patients are still diagnosed at an advanced or terminal stage (see Table 6) [58].

While a portion of the population recognises chronic hepatitis as a risk factor for liver cancer, awareness of MASLD as a risk factor for HCC remains low, limiting early intervention.

Preventing hepatitis infections and reducing metabolic risk factors are crucial for reducing HCC incidence, yet MASLD management plans require improvement, as lack a structured approach to address lifestyle modifications and fibrosis progression. Furthermore, many patients are unaware of the strong association between liver fibrosis severity and HCC occurrence [60]. Without reimbursement for serological and ultrasound-based non-invasive fibrosis tests, these tests cannot be effectively incorporated into high-risk patient identification for individuals with resolved HBV, cured HCV, habitual alcohol consumption, or MASLD.

Surveillance relies primarily on ultrasound and AFP, with limited use of PIVKA-II, which is only reimbursed for cirrhosis patients [61] and after curative treatment. Despite reimbursement for standard surveillance, poor patient adherence to surveillance [61] further delays diagnosis.

Broader use and reimbursement of PIVKA-II could improve early detection and reduce costly late-stage diagnoses [62]. Meanwhile, immunotherapy reimbursement for intermediate and advanced HCC is limited to one-time eligibility, restricting patient access to ongoing treatment. Expanding reimbursement policies and involving more patient advocacy in reimbursement decisions could help ensure broader, more sustainable access to essential therapies.

In Thailand, limited up-to-date knowledge of HCC among general practitioners hampers early symptom recognition and timely referral to specialised care [63]. As HCC aetiologies evolve from viral to metabolic and toxicological causes, awareness among the Thai population must adapt. Although ongoing efforts to improve awareness, a more consistent and unified public health policy is needed to address these knowledge gaps, enabling earlier diagnosis and more effective, less expensive treatment (see Table 7).

Early detection in high-risk adults remains inadequate. Without a comprehensive and consistent approach to identifying at-risk individuals, many cases go undiagnosed until advanced stages, leading to poorer patient outcomes and increased healthcare costs. A major challenge in HCC surveillance is the lack of a unified national database for hepatitis and HCC, hindering effective surveillance and timely interventions.

In Thailand, HCC surveillance relies primarily on sonography and AFP. Ultrasound is operator-dependent, and the country faces a shortage of trained sonographers, particularly in rural areas. As a result, many patients struggle to access routine surveillance, delaying diagnoses. The AFP biomarker, despite its widespread use, has limited sensitivity in early-stage HCC, further contributing to missed diagnoses [64]. Additionally, promising digital algorithms for early detection, such as GAAD/GALAD, are neither reimbursed nor integrated into the healthcare system. This, combined with limited diagnostic options, exacerbates Thailand’s challenges in improving HCC surveillance and early intervention.

Recent cost-effectiveness analyses show that blood-based biomarker screening using GAAD, GALAD, and PIVKA II +AFP outperforms the current US + AFP standard, improving early detection and survival [65]. Biomarker-based surveillance is also cost-effective when conducted biannually [65].

Access to treatment is another concern. In Thailand, reimbursement for systemic therapies and treatments such as Radiofrequency ablation for early-stage HCC is inconsistent and not always covered under the Universal Health Coverage or Social Security Scheme [66]. As a result, only 7% of the population under the Civil Servant Medical Benefit Scheme have access to these options, highlighting a significant gap in equitable treatment accessibility [67].

A major obstacle in addressing HCC is low public awareness. HCC is not widely recognised, and its risk factors are often underestimated, especially in rural areas where health education is limited [68]. This leads to delayed medical consultation and diagnosis, with many cases identified at later stages when treatment is less effective and more expensive (see Table 8) [68].

Access to preventive measures like vaccination and testing is also limited. Although HBV vaccination is part of the National Immunisation Programme, its reach in rural and underserved areas is inadequate [68]. Hepatitis C testing and treatment are not widely integrated into the healthcare system, leaving many unaware of their infection and at risk of developing HCC [69].

Timely and accurate diagnosis of HCC is hindered by a shortage of trained professionals and diagnostic equipment, particularly in rural areas. Specialists are concentrated in urban centres, delaying access for patients in remote regions. The limited availability of advanced diagnostic technologies further complicates early detection.

Finally, access to advanced treatments for HCC is restricted by regulatory and insurance barriers, and a lack of specialists in provincial hospitals [70]. As a result, patients from rural areas travel long distances to major hospitals for treatment, further straining the healthcare system. This inequitable resource distribution deepens disparities in health outcomes across the country.

## 5. Actionable Recommendations for Hepatocellular Carcinoma Surveillance and Management in the Asia Pacific

Using Japan’s HCC surveillance and management approach as a gold standard, local experts proposed actionable solutions for specific challenges along the patient journey, assigning implementation responsibilities to relevant agencies. These are summarised in the tables below.

While all solutions are important, it is essential to adopt a stepwise approach, prioritising solutions with the greatest health and economic impact. Preventing hepatitis and non-viral risk factors, such as MASLD, and expanding HCC surveillance are cost-effective strategies that help prevent HCC or enable early treatment [62,65,71,72,73].

Effective HCC policy must also account for differences in resources, surveillance, diagnostics, and access to curative treatment across health systems. For instance, where efforts to expand and drive uptake of HCC surveillance are already underway, priority should be given to identifying and eliminating implementation barriers, and addressing any challenges associated with diagnosis and treatment access to maximise both health outcomes and economic benefits.

These solutions, mapped to each health system’s specific challenges and resource constraints, offer tangible benefits for diverse stakeholders. For policymakers, they provide actionable insights and a clear framework to guide decision-making, allocate resources effectively, and foster collaboration. For patients and communities, they enhance access to timely detection, diagnosis, and treatment, improving quality of life and reducing the financial strain of care. From an economic perspective, prioritising prevention and early intervention can lower healthcare costs, boost workforce productivity, and contribute to sustainable public health outcomes.

The Roadmap to Liver Cancer Control in Australia by the Cancer Council identified Indigenous and CALD populations as high-risk groups for HCC, emphasising the disproportionate burden of liver cancer within these communities and the need for targeted interventions [74]. A co-designed, culturally appropriate approach using the “Double Diamond” framework that actively involves these communities is crucial in creating healthcare systems judged to be safe to approach and use, contributing to raising awareness and preventing HCC (see Table 9) [75,76].

Despite the relatively high alcohol use in Australia, local evidence on alcohol-related mortality is limited. However, given the harms caused by alcohol, a comprehensive approach to reducing alcohol use is essential to lower the risk of alcohol-induced cirrhosis, a key HCC contributor [77,78]. Additionally, expanding HBV and HCV surveillance and ensuring timely diagnosis and treatment are also critical. For instance, early antiviral therapy can significantly reduce HCC risk among individuals with chronic HBV [79]. Enhanced primary care education on the link between diabetes, obesity and HCC also aligns with the Australian National Diabetes Strategy’s goal of promoting awareness and earlier detection of diabetes through primary care providers [80].

Current HCC surveillance primarily relies on ultrasound and AFP [28], but emerging evidence in Thailand, the United Kingdom and China supports the incremental integration of novel biomarkers such as PIVKA-II and digital algorithms like GAAD, which are cost-effective and improve early detection [65,71,72]. Additionally, given the emergence of MASLD as a leading HCC risk factor in Australia [81], the national surveillance programme must evolve to include MASLD patients. To address the limitations of ultrasound accuracy, particularly in obese patients, AI-based screening programmes for HCC can be considered to reduce diagnostic variability and enhance the accuracy of HCC detection [82].

As per the Roadmap to Liver Cancer Control, experts recommend implementing a national HCC surveillance programme in the next five years [74], similar to Japan and South Korea. Japan’s national surveillance programme enables early detection in 68% of HCC cases [19], which is critical, as early diagnosis allows curative treatments such as surgical resection, liver transplantation, or ablation [83].

Once awareness, prevention and early detection are strengthened, the focus can shift to ensuring equitable access to diagnosis and treatment. The Optimal Care Pathway for HCC by Cancer Australia and the Cancer Council [84] supports the implementation of the national Australian Cancer Plan to achieve equity, access, and cultural sensitivity. For instance, improving access to multidisciplinary teams improves survival rates and uptake of curative treatment by facilitating early specialist input, optimising treatment pathways, and ensuring appropriate allocation of curative therapies [85,86]. Comprehensive cancer care networks that integrate general practitioners, gastroenterologists, hepatologists, oncologists, and hepato-pancreato-biliary surgeons will also facilitate timely diagnosis and treatment [84].

In India, efforts to improve awareness, prevention and early detection should be prioritised over the next 12–24 months (see Table 10). To improve awareness, existing counselling services should be scaled up by integrating them into broader health initiatives. This includes training counsellors to incorporate integrated health priorities–such as promoting behaviour change to combat obesity, alcohol consumption, and diabetes–into their programmes, driving cost reduction while improving health outcomes [87].

Expanding counselling programmes could also align with behavioural change strategies, as awareness alone is insufficient for long-term disease prevention, including conditions associated with obesity and diabetes [88], which are HCC risk factors. Successful public health interventions must go beyond awareness and incorporate behaviour-changing strategies, achieved through such counsellor-led programmes and broader public campaigns. While counsellors provide personalised support, large-scale campaigns using social media, community outreach, and workplace education can ensure wider reach and engagement.

For prevention, the National Viral Hepatitis Control Programme aims to ensure injection safety by formulating a policy to use Re-Use Prevention (RUP) syringes [36]. Scaling up affordable RUP syringe use and safe injection practices supports the effective implementation of this policy and reduces the hepatitis transmission risks.

Additionally, addressing the social determinants of health–including access to preventive measures–is important in designing effective HBV interventions [89]. Health authorities should ensure that affordable preventive services, such as vaccinations and infection control practices, are accessible in both urban and rural areas.

Regarding early detection and diagnosis, refining surveillance by integrating novel biomarkers like PIVKA-II and newer algorithms like GAAD can improve early detection [41]. Shifting from hospital-based imaging to community-based approaches using blood markers and digital technologies could be an effective solution for reaching at-risk populations [41]. Such advancements could significantly improve the precision and timeliness of HCC diagnosis. [62,65,71,72,73].

After improving awareness, prevention and early detection, the final challenge is ensuring that HCC treatments are accessible and affordable. Key recommendations include working with the government to develop sustainable financial models that ensure the affordability of life-saving treatments for HCC, such as liver transplantation and advanced chemotherapy. These include blending financing, which is the strategic use of development funds, such as those from government aid and philanthropic sources, to catalyse and mobilise private capital [13]. Blended financing has successfully augmented domestic financing and scaled up hepatitis health programmes [13]. Similar models can be explored for HCC.

Incentives for research and development (R&D) through government partnerships are essential for creating an attractive treatment R&D environment. For instance, the U.S. Cancer Moonshot Initiative, which accelerates research by providing funding and fostering public-private partnerships, has facilitated breakthroughs in immunotherapy and new precision medicine [90]. India has launched a similar initiative with the support of the U.S. In 2025, the U.S. FDA’s Oncology Centre of Excellence will visit India to set up collaborations under ‘Project Asha’ [91]. This partnership will focus on capacity-building, clinical trials, regulatory expertise, and increasing cancer clinical trial access [91].

In terms of awareness, implementing advocacy programmes targeting policymakers and healthcare professionals outside of hepatology and gastroenterology is key (see Table 11). Nationwide awareness campaigns can be launched to educate primary care physicians, laboratory personnel, and high-risk populations about HCC risks, early signs, and the importance of timely surveillance [45]. Additionally, strengthening Malaysia’s national cancer registry will provide more accurate data to inform public health initiatives and resource allocation.

For prevention, integrating HCC risk factor assessments into existing noncommunicable disease surveillance programmes will allow for earlier intervention. By embedding liver disease surveillance and stratification, particularly for HBV, HCV and MASLD, into routine healthcare visits, primary care providers can detect and manage risk factors before they progress to liver cancer. This strategy aligns with Malaysia’s existing public health infrastructure and can be scaled efficiently by leveraging digital health tools and electronic medical records.

With early detection being critical to improving survival rates, the establishment of surveillance programmes to identify high-risk individuals and ensure timely referrals is essential. This can be achieved by integrating electronic medical records that flag patients with known HCC risk factors, prompting physicians to conduct necessary surveillance. Additionally, expanding access to non-invasive surveillance modalities in tertiary centres and primary healthcare settings will help detect liver cancer at an earlier, more treatable stage.

Regarding diagnosis and access to treatment, strengthening multidisciplinary team approaches is crucial. Establishing minimum requirements using the Extension for Community Healthcare Outcomes model, which includes virtual multidisciplinary teams with interventional radiologists, gastroenterologists, hepatologists, and oncologists, will improve diagnostic accuracy and streamline treatment planning. Moreover, advocating for a value-based approach to healthcare where treatments are selected based on their effectiveness, cost, and accessibility, can help policymakers prioritise investments. Similarly, establishing a structured framework to enhance patient group participation in reimbursement decision-making, increasing awareness of the economic and social aspects of HCC, and advocating for a stronger patient voice in treatment reimbursement policies can help improve overall access to treatment.

For awareness, the priority is to expand existing public awareness campaigns led by the Korean Liver Cancer Association (see Table 12). While there have been efforts to inform the public, these programmes can be optimised to target high-risk individuals and promote early detection. Cost-effective strategies such as media campaigns, community outreach, and integrating awareness into primary care settings can quickly and effectively reach a large population, including high-risk groups [92]. This approach, which builds on existing frameworks, ensures maximum impact without substantial financial investments.

In terms of prevention, South Korea has laid a strong foundation with its 2017 national HCV surveillance programme [21], further updated in 2024 to expand coverage and improve early identification [93]. By further strengthening and implementing this updated policy, South Korea can achieve better health outcomes and generate significant economic savings.

For early detection, the national HCC guidelines should be updated to reflect emerging evidence on multi-biomarker approaches for early diagnosis. Current guidelines rely solely on AFP and PIVKA II, which have limitations, especially in detecting early-stage HCC [53,54]. Incorporating additional biomarkers, such as AFP-L3, and using them in combination could significantly enhance early detection [21]. This solution is feasible and cost-effective, as advancements in biomarker research allow guideline updates within the next 12 to 24 months with minimal additional costs.

While the solutions for awareness, prevention, and early detection are immediate and cost-effective, diagnosis and access to treatment will require more systemic changes and may take longer to implement. In particular, expanding reimbursement policies to improve patient access to essential HCC treatments is critical and will require close collaboration between the National Health Insurance Service, government agencies, pharmaceutical companies, and academic societies. Although this will require more time and coordination, these changes are essential to ensure equitable access to timely and effective treatments.

In Taiwan, a more targeted and proactive approach to health communication is needed to improve awareness of the importance of regular screening and early intervention (see Table 13). Digital platforms such as mobile applications can provide personalised education. Workplace-based health checkups and educational outreach would further enhance engagement, especially among working-age individuals who may not actively seek medical attention^85^. Cross-agency collaboration through standardised data-sharing platforms, such as Fast Healthcare Interoperability Resources systems, can facilitate more effective public health messaging and outreach, ensuring that at-risk populations receive the information necessary to make informed healthcare decisions [94].

Expanding prevention efforts is also critical. While Taiwan has made notable progress in eliminating hepatitis-related HCC, addressing metabolic and lifestyle-related risk factors is needed. A comprehensive metabolic syndrome management initiative that promotes exercise, dietary modifications, and proactive risk assessment would help mitigate these risks. Digital engagement through social media and gamified health education programmes could encourage participation in preventive measures, making health literacy more accessible and interactive [95]. Additionally, integrating predictive risk models into routine healthcare checkups would enable earlier identification of high-risk individuals and more tailored prevention strategies [96]. Encouraging insurers to offer incentives for preventive health behaviours, such as reduced premiums for those actively engaging in lifestyle modifications, could further support a culture of long-term health awareness and disease prevention.

Enhancing early detection and surveillance adherence is key to identifying HCC at more treatable stages. Improving risk stratification through a high-risk patient calculator, combining ultrasound, AFP and PIVKA-II, could help with resource allocation and ensure early detection of high-risk individuals. Recent research indicates that integrating the GAAD algorithm (gender, age, AFP, and PIVKA-II) into surveillance strategies may enhance detection accuracy and clinical effectiveness [64,65,97]. Additionally, a cost-effectiveness analysis is currently underway in Taiwan to determine the optimal surveillance strategy that balances cost and early detection benefits, further emphasising the need to optimise HCC surveillance for high-risk populations.

While diagnosis and access to treatment are crucial areas for long-term consideration, they require greater financial investment and regulatory changes. In the future, efforts to improve access to advanced diagnostic tools and systemic therapies and enhance patient advocacy in reimbursement decisions can be explored. Addressing limited reimbursement policies for immunotherapy and systemic treatments could help ensure that patients diagnosed at later stages have broader access to life-extending therapies. Similarly, establishing a structured framework to enhance patient group participation in reimbursement decision-making, increase awareness of economic and social aspects of HCC, and advocate for a stronger patient voice in treatment reimbursement policies can help to improve overall access to treatment.

At the awareness stage, addressing knowledge gaps among GPs and the younger population is crucial, especially with the shift in HCC causes towards metabolic and toxic risk factors in Thailand (see Table 14). Training programmes should be implemented in medical schools to educate young healthcare professionals, ensuring they can identify risk factors early. Expanding public awareness through social media campaigns is also essential to inform the younger population about risk factors and prevention. At the 2024 HCC APAC Policy Forum, hosted by the APAC Liver Disease Alliance, there was a strong emphasis on peer-to-peer advocacy, public awareness campaigns, and improved access to information to empower patients in making informed decisions [17]. A consistent government policy on HCC awareness could further enhance these efforts. This comprehensive approach could improve early detection, prevent disease progression, and reduce costs associated with advanced-stage treatments and hospitalisations.

In prevention, full reimbursement for HBV viral load testing is essential to ensuring patient access to treatment and reducing HCC incidence [17]. Increasing the number of hepatitis-related clinics, particularly at the local and community levels, would help address regional healthcare disparities and ensure high-risk individuals receive timely care. Encouraging routine surveillance can help identify early liver damage before it progresses to cancer. Through increased training, GPs would be able to recognise at-risk patients and implement preventive measures. By enhancing prevention and surveillance efforts, Thailand can reduce HCC rates and avoid the higher healthcare costs associated with advanced-stage liver cancer.

For early detection, expanding the use of novel biomarkers in surveillance programmes could improve the HCC detection accuracy at earlier stages, allowing for curative treatments like surgery or liver transplantation, which are more cost-effective than treating advanced-stage HCC. At the 2024 HCC APAC Policy Forum, the concept of liquid biopsies was introduced, which combines biomarkers such as PIVKA II and AFP-L3 with AFP and patient risk factors [17]. Models like the GALAD and AFP algorithms have shown promising results in early HCC detection. A study demonstrated that the GALAD score had a sensitivity of 70% and a specificity of over 90% for detecting early-stage HCC in a prospective, multicentre cohort [98]. The GAAD algorithm also demonstrates strong diagnostic performance, achieving an accuracy of 94.8% for all-stage HCC patients with chronic liver disease [99].

Combining biomarkers with clinical data could significantly enhance HCC surveillance and improve early detection. A comprehensive national database would also support better tracking of high-risk populations and provide data that could help shape effective prevention and treatment strategies [17]. Additionally, increasing access to surveillance programmes at the local and community levels would help ensure that high-risk individuals receive timely care.

Addressing diagnostic and access to treatment challenges in Thailand requires collaborative solutions, along with significant time and resources. Despite these challenges, Thailand has made significant progress through the Ministry of Public Health’s (MoPH) comprehensive efforts to enhance early detection and improve access to care. To promote early diagnosis, HBV and HCV screening programmes have been implemented, focusing on high-risk groups and individuals born before 1992, who missed hepatitis B vaccination prior to the national immunisation programme. Patients testing positive receive viral load testing, and patients diagnosed with HBV or HCV are treated according to national guidelines to ensure timely care.

The MoPH is also expanding public health insurance coverage and healthcare infrastructure. A nationwide laboratory testing and patient referral system is now in place, including hepatitis clinics at secondary care hospitals. Over 60% of GPs have completed online training to improve referrals.

To further improve diagnosis and treatment, reimbursement policies should cover diagnostic tests and treatments like radiofrequency ablation and systemic therapies, under the Universal Health Coverage scheme. Government support for affordable surveillance and treatment, along with reimbursement assistance for price-sensitive patients, would also help improve early diagnosis and timely access to care.

Improving public awareness is a high priority and can be achieved through tailored campaigns targeting different population groups, using channels like social media, community health centres, and local institutions (see Table 15). These efforts can help educate the public about the risks of HCC, particularly the connections with HBV, HCV and non-viral risk factors such as MASLD. By raising awareness, individuals are more likely to seek early surveillance and medical consultations, thereby reducing the burden of advanced-stage diagnoses.

In terms of prevention, expanding HBV vaccination coverage to underserved communities and promoting routine HCV testing are essential. A national policy on HCC prevention, supported by funding from organisations, would help strengthen these efforts.

Early detection is equally vital. Research into the cost-effectiveness of HCC surveillance programmes will demonstrate the value of driving the uptake of regular surveillance. Establishing standardised surveillance guidelines and enhancing healthcare infrastructure will allow for the early detection of HCC, which significantly increases treatment success rates and reduces overall healthcare costs. Additionally, the adoption of a national HCC surveillance programme that combines the use of AFP and PIVKA-II, alongside diagnostic algorithms like GAAD, would be both cost-effective and practical in the short term. This phased approach ensures a more gradual implementation, with the potential for greater success in early detection and better patient outcomes. Regular assessments of emerging biomarkers and diagnostic technologies can also be conducted to align with the latest scientific advancements. In addition, there should also be an inclusion of HCC surveillance and surveillance services within the national health insurance scheme. This would facilitate early detection, prevent the progression of the disease, and reduce long-term healthcare costs.

While solutions for diagnosis and treatment access are equally important, they are better addressed in the long term, as they require more resources. The shortage of trained specialists, diagnostic tools, and access to advanced treatment centres is a critical barrier that will take time to resolve. Nonetheless, the expansion of diagnostic capacity, particularly in rural areas, and the integration of more sophisticated diagnostic technologies will be necessary. Over time, the healthcare system should focus on enhancing diagnostic accuracy and increasing access to curative treatment options through increased sustainable reimbursement, which will further improve patient outcomes and reduce the economic burden on the system.

## 6. Turning Roadmaps into Action for Hepatocellular Carcinoma Surveillance and Management in the Asia Pacific

This policy article has highlighted the pressing need for a comprehensive and targeted response to HCC surveillance and management across the APAC region. Implementing the proposed recommendations requires dedicated efforts to secure necessary resources, ensuring sustainable and scalable solutions tailored to the unique needs of each healthcare system.

Effective collaboration among governments, healthcare providers, industry stakeholders, and patient advocacy groups is critical to driving meaningful progress. A united approach will help harmonise policies, leverage collective expertise, and optimise resource allocation to address the challenges associated with HCC surveillance and management. Aligning efforts with national health priorities will pave the way for impactful and sustainable change.

To translate national HCC roadmaps into tangible outcomes, stakeholders must move from planning to decisive action. Establishing clear governance structures, setting measurable targets, and implementing robust monitoring mechanisms will be key. Transparent progress tracking will enable stakeholders to evaluate the effectiveness of implemented strategies and ensure continuous refinement and improvement.

However, results will not appear overnight, as seen in Japan’s experience. Effective implementation requires sustained leadership and adaptability. Initial surges in detection may strain testing and treatment capacity, but long-term success depends on incremental improvements. Policymakers should anticipate these challenges and align resources accordingly to ensure a sustainable impact.

The ambition for APAC is to achieve significant advancements in HCC surveillance, diagnosis, and management, ultimately reducing mortality rates and improving quality of life. Drawing inspiration from world-leading models, such as Japan’s comprehensive approach to tackling HCC, can provide valuable insights for the region.

With a shared commitment and sustained engagement, each health system in the APAC region can make significant strides in reducing the burden of HCC, enhancing patient outcomes, and achieving long-term health and economic benefits. Establishing expert committees at the national level, involving all relevant stakeholders, will be instrumental in steering planning and implementation efforts effectively within each health system.

As HCC continues to pose a growing public health challenge, the urgency to act cannot be overstated. Every stakeholder in the ecosystem, from Ministries of Health, policymakers and funders, industry and providers, or physicians, caregivers and patients, has something to offer and something to gain. Through collective efforts, the region can move closer to a future where early detection, timely treatment, and comprehensive care are accessible to all.

## 7. Conclusions

Addressing HCC in the Asia-Pacific requires urgent, tailored action. These roadmaps present clear, evidence-based recommendations–rooted in regional insights and proven models–that each health system can adapt to its unique context. Prioritising awareness, prevention, advanced surveillance, and equitable treatment access will significantly improve patient outcomes and reduce economic burden.

Success depends on committed collaboration among policymakers, healthcare providers, and stakeholders, supported by clear governance and measurable targets. By moving swiftly from planning to implementation, the region can ensure that early detection and timely treatment are accessible to all, ultimately transforming the future of HCC care.

## Figures and Tables

**Table 1:** Japan’s roadmap outlining its comprehensive and structured approach to HCC surveillance and management.

PATIENT JOURNEY STAGE	CHALLENGE	POTENTIAL SOLUTION(S)
**AWARENESS**	• Increasing difficulty in defining high-risk non-viral HCC patients and the need for broader awareness of emerging conditions like MASLD	• **“Stop HCC” campaign (1995)**: Educates general practitioners (GP) and the public on early detection and treatment of HCC every year since 1995 • **Annual public awareness campaigns**: Focus on the increased knowledge of high-risk populations for HCC, importance of early detection and proactive surveillance • **Designated leaders**: Each of Japan’s 47 prefectures has a leader for hepatology education, nominated by Japan Society of Hepatology
**PREVENTION**	• Reducing HCC cases through preventive measures	• **2009 Basic Act on hepatitis measures** [15]: Provides free universal testing for HBV and HCV at public health clinics or all the clinics/hospitals • **Universal HBV vaccination (2016):** Universal vaccination programme was launched in 2016 to prevent mother-to-child-transmission (MTCT) of the virus • **Special subsidy programmes [15]**: Cover antiviral treatment for HBV and HCV patients
**EARLY DETECTION**	• Ensuring that surveillance systems remain effective as the characteristics of HCC patients change (e.g., non-viral causes)	• **Surveillance for HCC in at-risk patients is covered by the national insurance:** Alpha-Fetoprotein (AFP), Protein Induced by Vitamin K Absence or Antagonist-II (PIVKA-II), Lectin-reactive Fraction of Alpha-fetoprotein (AFP-L3), ultrasound, Computed Tomography (CT), Magnetic Resonance Imaging (MRI) for hepatitis, MASLD and cirrhosis are all reimbursed • **Advanced surveillance modalities**: Early adoption of PIVKA-II (1989) and AFP-L3 (1994), with simultaneous testing along with AFP encouraged and offered at a cost-effective rate • **Surveillance recommendations**: Ultrasound and 3 tumour markers every 6 months for high-risk patients; every 3–4 months for extremely high-risk patients, with optional dynamic CT/MRI
**DIAGNOSIS**	• Accurate and timely diagnosis of HCC, particularly in patients with complex or less common conditions	• **Advanced imaging coverage:** Includes dynamic CT, MRI, contrast-enhanced ultrasound, or contrast-enhanced MRI for cases where ultrasound is inadequate • **Referral system:** Electronic medical records prompt specialist referrals based on positive viral hepatitis tests
**ACCESS TO TREATMENT**	• Ensuring that patients have access to necessary treatments and follow-up care	• **Special subsidy programmes [16]:** Provide financial support for treatments for hepatitis, and a subsidy programme for treatment of liver cancer and decompensated cirrhosis (only for patients with HBV or HCV infection, not for non-viral cirrhosis/HCC) • **Integrated surveillance system and easy access programme to sophisticated treatment:** Ensures that surveillance and treatment services are accessible across community hospitals, GPs, and small clinics, with referral systems to larger centres for specialised care, including locoregional therapy and systemic therapy, all of which are covered by insurance along with special coverage system for high-cost treatment, such as immunotherapy, by government

**Table 2:** HCC challenges in Australia.

PATIENT JOURNEY STAGE	CHALLENGE
**AWARENESS**	• Needs of indigenous and culturally and linguistically diverse (CALD) groups are not addressed • High prevalence of diabetes and obesity risk factors
**PREVENTION**	• High alcohol consumption rates • Viral hepatitis is not always diagnosed and treated in a timely manner
**EARLY DETECTION**	• Suboptimal access to ultrasound surveillance services and primary care diagnosis capabilities • Limitations in ultrasound accuracy for obese patients
**DIAGNOSIS**	• Suboptimal access to MRI for accurate diagnosis • Patient support services could be better
**ACCESS TO TREATMENT**	• Geographical and equity-related disparities • Lack of funded 2nd-line systemic therapies • Lack of management of HCC by non-oncologists

**Table 3:** HCC challenges in India.

PATIENT JOURNEY STAGE	CHALLENGE
**AWARENESS**	• Lack of effective communication to scale up awareness [38] • Despite high awareness of the risks, behavioural change remains a challenge, with persistently high prevalence of diabetes, obesity-related risk factors, and excessive alcohol consumption [39]
**PREVENTION**	• Lack of affordable RUP (Re-Use Prevention syringes) [40] • Large geographic diversity with difficulties in reaching isolated and disadvantaged patients [37]
**EARLY DETECTION/DIAGNOSIS**	• HCC surveillance is neither well-organised nor universally practised [41]
**ACCESS TO TREATMENT**	• Many treatment modalities are not accessible or affordable for a significant portion of the patient population [35]

**Table 5:** HCC challenges in South Korea.

PATIENT JOURNEY STAGE	CHALLENGE
**AWARENESS**	• Limited public awareness activities related to HCC prevention and early detection
**PREVENTION**	• Before the national HCV surveillance programme, many opportunities for early intervention were missed. While detection has improved since its launch, gaps persist
**EARLY DETECTION/DIAGNOSIS**	• Current HCC surveillance guidelines rely on AFP as the only biomarker, which may limit the accuracy of early detection
**ACCESS TO TREATMENT**	• Limited reimbursement options hinder patient access to essential HCC treatments

**Table 6:** HCC challenges in Taiwan.

PATIENT JOURNEY STAGE	CHALLENGE
**AWARENESS**	• Awareness that chronic hepatitis is a risk factor for HCC remains limited, and even fewer people recognise that MASLD is also a significant risk factor
**PREVENTION**	• Management plans for MASLD patients need enhancement to ensure better awareness and mitigation of HCC risk • Patients with resolved HBV, MASLD, and alcoholic liver disease at risk of fibrosis require emphasis on fibrosis evaluation, which may be often overlooked
**EARLY DETECTION/DIAGNOSIS**	• Surveillance relies on ultrasound and AFP, with limited use of PIVKA-II which restricts more accurate and early detection of HCC
**ACCESS TO TREATMENT**	• Immunotherapy reimbursement for intermediate/advanced HCC cases has a one-time eligibility • Patient advocacy involvement in reimbursement decisions could be more active

**Table 7:** HCC challenges in Thailand.

PATIENT JOURNEY STAGE	CHALLENGE
**AWARENESS**	• Insufficient updated knowledge among GPs, particularly regarding the rising prevalence of metabolic and toxic risk factors • Low awareness among the general population • Lack of continuity in government policy
**PREVENTION**	• HBV Viral load is underutilised due to the budget constraints of capitation reimbursement, restricting access for high-risk patients needing hepatitis treatment and HCC surveillance
**EARLY DETECTION/DIAGNOSIS**	• Early detection in high-risk adults is inadequate • Lack of a unified national database for hepatitis and HCC, posing challenges for tracking and surveillance • The HCC surveillance programme using ultrasound and AFP is inadequate, especially in resource-limited settings • New blood-based biomarkers for HCC surveillance, such as PIVKA II, which have the potential to improve accessibility and accuracy at the national level, are currently not included in policy agendas or reimbursement programmes
**ACCESS TO TREATMENT**	• Radiofrequency ablation for early stage cannot be reimbursed under universal health coverage scheme • Systemic therapy cannot be reimbursed for advanced stages

**Table 8:** HCC challenges in Vietnam.

PATIENT JOURNEY STAGE	CHALLENGE
**AWARENESS**	• Community awareness about HCC is significantly lower compared to other diseases such as lung and breast cancer • The risk of HCC is often underestimated • There is a disparity in awareness levels between urban and rural areas
**PREVENTION**	• Although HBV is included in the National Immunisation Programme, some outreach communities have no access to it • HBV and HCV tests are not yet widely recognised as standard surveillance tools, resulting in limited testing and missed opportunities for early detection
**EARLY DETECTION**	• Surveillance for HCC is not covered by national health insurance • Lack of standardised surveillance guidelines • Insufficient healthcare workforce and infrastructure for surveillance (i.e., technology, risk classification systems)
**DIAGNOSIS**	• There are challenges in accurate and timely diagnostics due to an imbalance in workforce distribution, limited access to diagnostic technology, tools and services
**ACCESS TO TREATMENT**	• Advanced therapies are lacking due to regulatory and insurance coverage limitations • Limited access to HCC treatment centres, with severe and late-stage cases treated only in major hospitals • Shortage of specialists and treatment centres in provincial hospitals

**Table 9:** Overview of recommendations for addressing HCC key challenges in Australia.

PATIENT JOURNEY STAGE	CHALLENGE	POTENTIAL SOLUTION(S)	RESPONSIBLE AGENCY/DEPARTMENT
**AWARENESS**	• Needs of indigenous and culturally and linguistically diverse (CALD) groups are not addressed • High prevalence of diabetes and obesity risk factors	• Implement community co-design initiatives, leveraging the “Double Diamond” framework • Enhance primary care education	• Government (national, jurisdictional, cancer council)
**PREVENTION**	• High alcohol consumption rates • Viral hepatitis is not always diagnosed and treated in a timely manner	• Introduce alcohol control policies, incl. advertising regulations and minimum pricing • Implement “sugar” tax policies • Solutions should align with national HCV, HBV, obesity, and diabetes strategies, the 2023 Roadmap to Liver Cancer Control (2-, 5-, and 10-year goals), and the Australian Cancer Plan	
**EARLY DETECTION**	• Suboptimal access to ultrasound surveillance services and primary care diagnosis capabilities • Limitations in ultrasound accuracy for obese patients	• Expand comprehensive surveillance for HCV/HBV in all at-risk populations • Expand ultrasound surveillance programmes • Introduce blood-based biomarker tests i.e., GAAD/GALAD • Implement risk stratification tools • Strengthen primary care capacity to diagnose liver disease and establish referral pathways • Launch a national surveillance programme for high-risk groups, incl. individuals with cirrhosis and HBV	
**DIAGNOSIS**	• Suboptimal access to MRI/CT for accurate diagnosis • Patient support services could be better	• Optimise cost-effectiveness measures to improve access to diagnostic modalities (MRI/CT) and patient support services • Secure funding through the Medical Services Advisory Committee to enable access to diagnostics • Develop a comprehensive surveillance strategy • Expand the role of liver specialist nurses • Establish patient support lines and navigation services	
**ACCESS TO TREATMENT**	• Geographical and equity-related disparities • Lack of funded 2nd-line systemic therapies • Lack of management of HCC by non-oncologists	• Implement a national Australian cancer plan • Improve access to multidisciplinary teams • Develop comprehensive cancer care networks • Increase reimbursement and funding for second-line systemic therapies • Ensure that all solutions align with the 2023 HCC Surveillance Guidelines and Optimal Care Pathways	

**Table 10:** Overview of recommendations for addressing HCC key challenges in India.

PATIENT JOURNEY STAGE	CHALLENGE	POTENTIAL SOLUTION(S)	RESPONSIBLE AGENCY/DEPARTMENT
**AWARENESS**	• Lack of effective communication to scale up awareness [38] • Despite high awareness of the risks, behavioural change remains a challenge, with persistently high prevalence of diabetes, obesity-related risk factors, and excessive alcohol consumption [39]	• Scale up the existing counselling services (integrated for efficiency) to include counsellors trained to address integrated health priorities/programmes, driving efficiency and cost reduction	• Community health centre, led by a Chief Medical Officer at the block level, operating under the jurisdiction of the district-level health administration
**PREVENTION**	• Lack of affordable RUP (Re-Use Prevention syringes) [40] • Large geographic diversity with difficulties in reaching isolated and disadvantaged patients [37]	• Continue scaling up injection safety projects at a national level [42] • Ensure a more sustained supply of HCV therapy by extending treatment availability beyond just a month, incorporating lessons learned from the COVID-19 pandemic	• National Viral Hepatitis Management Unit [42] • Community health centre, led by a Chief Medical Officer at the block level, operating under the jurisdiction of the district-level health administration
**EARLY DETECTION DIAGNOSIS**	• HCC surveillance is neither well-organised nor universally practised [41]	• Refine HCC surveillance strategies by integrating novel biomarkers like PIVKA II, and newer algorithms like GAAD/GALAD [41]	• Ministry of Health and Family Welfare
**ACCESS TO TREATMENT**	• Many treatment modalities are not accessible or affordable for a significant portion of the patient population [35]	• Work with government to develop sustainable financial models for affordable care • Develop incentives to enable an attractive treatment research and development (R&D) environment	• Ministry of Health and Family Welfare

**Table 11:** Overview of recommendations for addressing HCC key challenges in Malaysia.

PATIENT JOURNEY STAGE	CHALLENGE	POTENTIAL SOLUTION(S)	RESPONSIBLE AGENCY/DEPARTMENT
**AWARENESS**	• Low awareness among HCPs outside of hepatologists and gastroenterologists • Low awareness among potential at-risk patients, with a need to better identify who falls into high-risk categories • Low awareness among laboratory personnel about the availability of relevant tests	• Implement HCC advocacy programmes targeting policymakers, healthcare professionals, lab professionals and high-risk patients • Strengthen the national cancer registry	• Ministry of Health
**PREVENTION**	• Lack of screening for HCC risk factors (e.g., HBV, HCV, MASLD, and alcohol-related liver conditions) to prevent progression to HCC	• Integrate HCC risk factor assessments into existing noncommunicable disease surveillance programmes	• Ministry of Health
**EARLY DETECTION**	• Lack of HCC surveillance for early detection of high-risk groups	• Establish optimal HCC surveillance programmes for high-risk individuals and ensure timely referrals, potentially integrating electronic medical records	• Ministry of Health
**DIAGNOSIS**	• Diagnoses and management are not conducted within a multidisciplinary team setting, hindering access to care and treatment • Access to a multidisciplinary team approach needs to be strengthened (e.g., virtual multidisciplinary team discussion, to physically refer if there is a definitive management plan)	• Establish minimum requirements using the Extension for Community Healthcare Outcomes model, including virtual multidisciplinary teams comprising interventional radiologists, gastroenterologists, hepatologists, hepatobiliary surgeons and oncologists • Advocate for a value-based approach using a template model to guide policymakers in accessing evidence-based treatments	• Ministry of Health
**ACCESS TO TREATMENT**	• Lack of access to evidence-based treatment options for patients		

**Table 12:** Overview of recommendations for addressing HCC key challenges in South Korea.

PATIENT JOURNEY STAGE	CHALLENGE	POTENTIAL SOLUTION(S)	RESPONSIBLE AGENCY/DEPARTMENT
**AWARENESS**	• Limited public awareness activities related to HCC prevention and early detection	• Leverage existing awareness programmes led by the Korean Liver Cancer Association • Introduce targeted government initiatives to enhance public knowledge and engagement	• Ministry of Health and Welfare
**PREVENTION**	• Before the national HCV surveillance programme, many opportunities for early intervention were missed. While detection has improved since its launch, gaps persist	• Implement a new national surveillance policy/programme for HCV, launched in 2024 to ensure wider coverage and early detection of HCC risk factors	• Ministry of Health and Welfare • Korea Disease Control and Prevention Agency
**EARLY DETECTION/DIAGNOSIS**	• Current HCC surveillance guidelines rely on AFP as the only biomarker, which may limit the accuracy of early detection	• Update national HCC guidelines to incorporate emerging evidence on multi-biomarker approaches and improve early detection rates	• Ministry of Health and Welfare
**ACCESS TO TREATMENT**	• Limited reimbursement options hinder patient access to essential HCC treatments	• Expand reimbursement policies through collaborative efforts involving the National Health Insurance Service, government agencies, pharmaceutical companies, and academic societies to enhance treatment accessibility	• National Health Insurance Service • Ministry of Health and Welfare

**Table 13:** Overview of recommendations for addressing HCC key challenges in Taiwan.

PATIENT JOURNEY STAGE	CHALLENGE	POTENTIAL SOLUTION(S)	RESPONSIBLE AGENCY/DEPARTMENT
**AWARENESS**	• Awareness that chronic hepatitis is a risk factor for HCC remains limited, and even fewer people recognise that MASLD is also a significant risk factor	• Deliver personalised health education via mobile apps, focusing on what patients should do rather than what they should avoid • Utilise social media to disseminate health information • Introduce gamification strategies, i.e., incentivised health education videos that reward users with points • Provide health information and checkups through workplaces • Enable cross-department data sharing (between Health Promotion Administration and National Health Insurance Administration) using standardised Fast Healthcare Interoperability Resources systems while ensuring privacy protection	• Health Promotion Administration (Ministry of Health and Welfare) • Ministry of Labor, Ministry of Defence, Ministry of Education
**PREVENTION**	• Management plans for MASLD patients need enhancement to ensure better awareness and mitigation of HCC risk • Patients with resolved HBV, MASLD, and alcoholic liver disease at risk of fibrosis require emphasis on fibrosis evaluation	• Introduce a comprehensive metabolic syndrome management plan or campaign, targeted at lifestyle interventions to reduce risk factors • Develop predictive risk models for at-risk MASLD patients • Promote health through exercise programmes and gym initiatives • Encourage social engagement through health-related activities • Offer spill-over insurance (i.e., policies that reward healthier lifestyle changes with reduced premiums) to promote healthier lifestyles	• Health Promotion Administration (Ministry of Health and Welfare)
**EARLY DETECTION/DIAGNOSIS**	• **Surveillance relies on ultrasound and AFP, with limited use of PIVKA-II which restricts more accurate and early detection of HCC**	• Implement targeted surveillance for cardiometabolic risk factors rather than general population surveillance • Enable the wider use of PIVKA-II for timely diagnosis by developing a high-risk patient calculator to optimise ultrasound, AFP, and PIVKA-II surveillance for cost-effective resource allocation.	• National Health Insurance Administration (Ministry of Health and Welfare)
**ACCESS TO TREATMENT**	• Immunotherapy reimbursement for intermediate/advanced HCC cases has a one-time eligibility • Patient advocacy involvement in reimbursement decisions could be more active	• Expand immunotherapy reimbursement beyond one-time eligibility to ensure sustained access to treatment • Establish a structured framework to enhance patient group participation in reimbursement decision-making, particularly in identifying treatments that should be considered for reimbursement • Increase awareness and engagement of patient groups to educate policymakers, regulatory bodies and healthcare payers on the economic and social impact of HCC, emphasising the importance of including patient perspectives in reimbursement decisions	• Taiwan’s Food and Drug Administration (Ministry of Health and Welfare) • National Health Insurance Administration (Ministry of Health and Welfare)

**Table 14:** Overview of recommendations for addressing HCC key challenges in Thailand.

PATIENT JOURNEY STAGE	CHALLENGE	POTENTIAL SOLUTION(S)	RESPONSIBLE AGENCY/DEPARTMENT
**AWARENESS**	• Insufficient updated knowledge among GPs, particularly regarding the rising prevalence of metabolic and toxic risk factors • Low awareness among the general population • Lack of continuity in government policy	• Implement training programmes in medical schools to educate young HCPs • Drive HCC as a national healthcare priority • Develop segmented social media campaigns combined with health education curriculum reform	• Department of Disease Control and Department of Medical Services, Ministry of Public Health of Thailand • Thai Health • Government • Consortium of Thai Medical Schools
**PREVENTION**	• HBV Viral load is underutilised due to the budget constraints of capitation reimbursement, restricting access for high-risk patients needing hepatitis treatment and HCC surveillance	• Optimise HBV Viral load budget in Universal Health Coverage reimbursement • Increase hepatitis-related clinics nationwide at local/community level to address regional disparities	• Government cabinet • Department of Disease Control, Ministry of Public Health of Thailand • National Health Security Office
**EARLY DETECTION/DIAGNOSIS**	• Early detection in high-risk adults is inadequate • Lack of a unified national database for hepatitis and HCC, posing challenges for tracking and surveillance • The HCC surveillance programme using ultrasound and AFP is inadequate, especially in resource-limited settings • New blood-based biomarkers for HCC surveillance, such as PIVKA II, which may enhance accessibility and accuracy, are not included in the policy agenda and reimbursement programmes	• Encourage surveillance in adults • Enhance the capacity of healthcare facilities to provide accurate diagnosis and effective treatment • Establish a structured system for laboratory testing and patient referral pathways • Emphasise greater focus on high-risk groups, such as cirrhosis patients, for surveillance programmes • Develop a unified comprehensive database for hepatitis and HCC to support early detection • Conduct a health technology assessment of new blood-based biomarkers for inclusion in national surveillance programmes • If the assessment demonstrates cost-effectiveness, reimburse new blood-based biomarkers for use in surveillance programmes, integrating both prevention and promotion strategies	• Department of Medical Services, Service plan, Ministry of Public Health of Thailand
**ACCESS TO TREATMENT**	• Radiofrequency ablation for early stage cannot be reimbursed under the universal health coverage scheme • Systemic therapy cannot be reimbursed for advanced stages	• Improve benefits in universal health coverage scheme • Include ablation needles in reimbursement in the universal health coverage scheme • Include systemic therapies under the universal health coverage scheme to increase access to care for advanced-stage HCC patients	• National List of Essential Medicines • National Health Security Office • Social Security Office

**Table 15:** Overview of recommendations for addressing HCC key challenges in Vietnam.

PATIENT JOURNEY STAGE	CHALLENGE	POTENTIAL SOLUTION(S)	RESPONSIBLE AGENCY/DEPARTMENT
**AWARENESS**	• Community awareness about HCC is significantly lower compared to lung and breast cancer • The risk of HCC is often underestimated • There is a disparity in awareness levels between urban and rural areas	• Enhance community awareness through tailored approaches targeting different population groups via social media, primary healthcare centres, and local community institutions such as wards	• Ministry of Health • Provincial health department • Commune health department
**PREVENTION**	• Although HBV is included in the National Immunisation Programme, some outreach communities have no access to it • HBV and HCV tests are not yet widely considered as universal surveillance tests	• Develop a national policy on HCC prevention, including vaccination and HBV/HCV testing • Call for funding from organisations to expand HBV vaccination outreach in underserved communities and integrate HBV and HCV testing as universal surveillance measures	• Centres for Disease Control and Prevention under the Ministry of Health
**EARLY DETECTION**	• Surveillance for HCC is not covered by NHI • Lack of standardised surveillance guidelines • Insufficient healthcare workforce and infrastructure for surveillance (i.e., technology, risk classification systems)	• Research the cost-effectiveness of surveillance programmes • Develop surveillance guidelines with clear recommendations • Implement capacity-building initiatives to enhance the healthcare workforce	• Ministry of Health
**DIAGNOSIS**	• There are challenges in accurate and timely diagnostics due to an imbalance in workforce distribution	• Focus capacity-building initiatives on enhancing diagnostic expertise, including ultrasound, CT, and MRI capabilities • Provide free diagnostic testing through government reimbursement programmes	• Ministry of Health
**ACCESS TO TREATMENT**	• Advanced therapies are lacking due to regulatory and insurance coverage limitations • Limited access to HCC treatment centres, with severe and late-stage cases treated only in major hospitals • Shortage of specialists and treatment centres in provincial hospitals	• Update HCC treatment guidelines regularly • Increase government subsidies to enhance access to treatment at lower-level hospitals, including provincial healthcare facilities • Explore sustainable reimbursement mechanisms to ensure long-term accessibility of innovative treatments • Implement capacity-building programmes to strengthen the healthcare workforce and address shortages	• Ministry of Health

## Data Availability

The original contributions presented in this study are included in the article. Further inquiries can be directed to the corresponding author.

## Abbreviations

AFP	Alpha-Fetoprotein
AFP-L3	Lectin-reactive Fraction of Alpha-fetoprotein
APAC	Asia Pacific
CALD	Culturally And Linguistically Diverse
CT	Computed Tomography
GAAD	Gender, Age, AFP, and Des-gamma carboxy-prothrombin (DCP)
GALAD	Gender, Age, AFP-L3, AFP, and Des-gamma carboxy-prothrombin (DCP)
GP	General Practitioner
HBV	Hepatitis B Virus
HCC	Hepatocellular Carcinoma
HCP	Healthcare Professional
HCV	Hepatitis C Virus
MASLD	Metabolic Dysfunction-associated Steatotic Liver Disease
MRI	Magnetic Resonance Imaging
PIVKA-II	Protein Induced by Vitamin K Absence or Antagonist-II
USD	United States Dollar
